# Complete genome of the mutualistic symbiont “*Candidatus* Carsonella ruddii” from a Japanese island strain of the Asian citrus psyllid *Diaphorina citri*

**DOI:** 10.1128/mra.01082-24

**Published:** 2025-02-25

**Authors:** Masaki Mizutani, Takashi Fujikawa, Takema Fukatsu, Shigeyuki Kakizawa

**Affiliations:** 1 Bioproduction Research Institute, National Institute of Advanced Industrial Science and Technology (AIST), Tsukuba, Japan; 2 Institute for Plant Protection, National Agriculture and Food Research Organization (NARO) 13516 , Tsukuba, Japan; 3 Department of Biological Sciences, Graduate School of Science, University of Tokyo, Tokyo, Japan; 4 Graduate School of Life and Environmental Sciences, University of Tsukuba 13121 , Tsukuba, Japan; University of Maryland School of Medicine, Baltimore, Maryland, USA

**Keywords:** insect endosymbiont, genome, PCR-amplified fragment

## Abstract

The complete genome, 173,958 bp in size, of “*Candidatus* Carsonella ruddii” DC-OKEB1*,* an obligate bacterial endosymbiont of the Asian citrus psyllid *Diaphorina citri*, was determined. The genome sequence provides valuable information for comparative and evolutionary aspects of the intimate insect–microbe mutualism.

## ANNOUNCEMENT

Mutualistic relationship with microbial symbiont is essential for growth and reproduction of various insect species, particularly those that depend on nutritionally deficient diets, such as plant sap (1). “*Candidatus* Carsonella ruddii” is a clade of essential symbiotic bacteria of psyllids, known for supplying essential amino acids to compensate for protein-deficient plant sap diet of the insect host (2). Their genomes are strikingly small, around 160 kbp with a limited number of genes, suggesting that they may have achieved organelle-like status (3–6). Despite the extreme genome reduction, the symbiont retains the capability of synthesizing several essential amino acids, reflecting its role in nutritional supplementation at the genomic level (2, 4). The Asian citrus psyllid, *Diaphorina citri* (Insecta: Hemiptera: Psylloidae), is a serious pest of citrus species worldwide, transmitting “*Candidatus* Liberibacter asiaticus,” the pathogen responsible for huanglongbing (citrus greening disease) (7). Due to the agricultural significance of the Asian citrus psyllid, extensive quarantine control measures are implemented globally, and interest in their physiology and ecology continues to grow.

Here, we sequenced the genome of “*Ca*. Carsonella ruddii” of the Asian citrus psyllid *D. citri* collected in China, Okinoerabu Island, Kagoshima, Japan (near the GPS location 27°20′17N 128°34′27E), which we designated as “*Ca*. Carsonella ruddii” DC-OKEB1. The genomic DNA was extracted from the insect body using NucleoSpin Food column (MACHEREY-NAGEL). DNA fragments covering the entire “*Ca*. Carsonella ruddii” genome were amplified by long-PCRs. In total, 15 primer sets were designed to amplify the entire 174 kbp genome of “*Ca*. Carsonella ruddii” DC (GenBank accession number CP003467), which was derived from *D. citri* collected in Amami-Oshima Island, Japan (5). Each primer was 35 nucleotides long (Table 1), and the amplified products ranged from 6 to 13 kbp in size. PCR amplifications were performed using KOD-One polymerase (TOYOBO) according to the manufacturer’s instructions. All the fragments were purified using PCR Purification Kit (QIAGEN). For library construction, the purified PCR products were fragmented and tagged using MGIEasy FS DNA Library Prep Set (MGI Tech), circularized using MGIEasy Circularization Kit (MGI Tech), and subjected to construction of DNA Nanoballs (DNBs) using DNBSEQ-G400RS High-throughput Sequencing Kit (MGI Tech). The DNBs were sequenced using DNBSEQ-G400 (MGI Tech) platform in a 150 bp paired-end mode. The obtained 12,081,706 paired reads were mapped onto the reference genome (GenBank: CP003467 and GCA_044336275.1) to check the quality of reads. The 8,359,808 mapped reads were applied to *de novo* assembly program using CLC Genomics Workbench 23.0.4 (QIAGEN). Default parameters were used for all software unless otherwise specified. The obtained contigs were manually assembled to construct a single circular genome with 7,206-fold coverage. For circularization, paired reads overlapping both contig ends were used, and the genome was rotated based on the reference genome. The constructed genome was polished by the read mapping using CLC Genomics Workbench and annotated using DFAST pipeline v1.6.0 (8).

**TABLE 1:** PCR primer pairs used in this study

Region	Amplified length (bp)	Forward primer name	Forward primer sequence(5′−3′)	Reverse primer name	Reverse primer sequence(5′−3′)
1	12,366	1-F	ATGAAAAATATTATTGTTGCAAAAGTTACTCCTGA	1-R2	ATAAATAAATCAGGTATTCCTATAATTATGTTTAG
2	12,458	2-F2	TAATGTGTTATTTAAAATAGGTAAAAATCCATACG	2-R	ACTACTAGATTATCAAATTTTTCTCTAGTAGATTC
3	12,500	3-F1	CAATTACCAAAAAAAAAATAAAAATAATTTTGTCT	3-R	AAAATAATAAATTTATATCCTATCATTAAAATCAA
4	12,781	4-F	AACGTAATAAAAATTGTGTAAATACTATTTTAATA	4-R2	TCTTTAATACATATCATTAAATGTTCAGGAGCAAG
5	12,852	5-F3	TTCGCTTTATTATATTATATAAAATTTATTGATGG	5-R2	TCTAAATCATTTAGTTTAGCTGGTTCACGTATAGG
6	13,045	6-F2	TTATAATGATTTGTACCAGGAGTGTTATTCTGAAT	6-R2	TTTACAAAATAATCTATTACGTCACTAATTAAATC
7	13,207	7-F2	TCTATAATAGGAAACGTTAATTCTGGTAAAACTAC	7-R3	TAAAATCCATCAATAATAGCACCAATGAGTAGACC
8	12,300	8-F1	AAAAAAAAAATTATTAATTTTTCTTTAAATGGTAT	8-R	CTCGATCAAGTAAATCTTTACAAATTAAAAAAAGA
9	12,651	9-F2	TTTAAAGTTGTAAAAATATTGTTCAATTTGAGGAG	9-R2	GCAATAATATATACAAAAACTACAGTTTATGCAAT
10	12,643	10-F2	AATCAAGAATTATTCCAATTAAAAAAGATAAATCT	10-R2	TACTGGAGCTCATAAAATGAATAATTCGATTGCTC
11	12,712	11-F3	TCCTGGATAATCTAATCCAGCTGATATCGAATTCG	11-R	AAGGAAGTGGAGTACCTGCTTTAATAGGTATTTAT
12	12,703	12-F	CAATTCTAACCTCCGCAAAAAATCAATTGACTATT	12-R	AGAAAAAATCAAATGATTGAATTTTATAAAAAATT
13	13,252	13-F5	AGATATGAAAATTTCCGATTCCCATAATTTATTAG	13-R	GTAACAACAAAAAATGTTTTTGTGATAAAACAAAC
14	13,034	14-F2	TTTAAATTAGGTCCAACAGAAATAACTGTTCTTCC	14-R2	GTAATTATGTCCTGATAAAGCTGCAGATCCTAAAG
15	6,095	15-F2	CCATACGAATAAATTGTAAATTAATATGTTGTTAA	15-R	TTTTATTTAAAAAAAAAAATTCCACTTGCCGAAAT

The complete circular genome of “*Ca*. Carsonella ruddii” DC-OKEB1 was 173,958 bp in size with 17.6% G + C content. It encoded putative 195 protein-coding sequences, 28 transfer RNAs, and a single copy of 16S/23S/5S ribosomal RNA operon. In comparison to the 174,014 bp genome of “*Ca*. Carsonella ruddii” DC (5), the genome sequences were highly similar but 23 nucleotide substitutions, and eight indels were identified (Table 2 at https://doi.org/10.6084/m9.figshare.28210259.v1). The amino acid synthesis pathways reported in “*Ca*. Carsonella ruddii” strains of *Diaphorina* spp. (5, 6) were identified in the results of the present genome analysis. These results are consistent with prior studies highlighting the nutritional role of “*Ca*. Carsonella ruddii” (2, 4). Our new symbiont genome data will contribute to comparative symbiont genomics that provide insight into how sophisticated insect–microbe mutualisms have evolved and diversified. Furthermore, the genome sequencing procedure adopted in this study, which involved amplification of the entire genomic regions by long-PCR prior to sequencing, will be useful for sequencing bacterial genomes for which only a tiny amount of DNA is available.

## Data Availability

The sequence data have been deposited to DDBJ under the accession numbers AP036056 for assembly and DRR591238 for raw reads. The results of sequence comparison between the determined genome and the reference genome are in Table 2 at https://doi.org/10.6084/m9.figshare.28210259.v1.
